# Heritable strategies for controlling insect vectors of disease

**DOI:** 10.1098/rstb.2013.0432

**Published:** 2014-06-19

**Authors:** Austin Burt

**Affiliations:** Department of Life Sciences, Imperial College London, Silwood Park, Ascot, Berks SL5 7PY, UK

**Keywords:** malaria, dengue, population genetic engineering, *Wolbachia*, RIDL, homing endonuclease genes

## Abstract

Mosquito-borne diseases are causing a substantial burden of mortality, morbidity and economic loss in many parts of the world, despite current control efforts, and new complementary approaches to controlling these diseases are needed. One promising class of new interventions under development involves the heritable modification of the mosquito by insertion of novel genes into the nucleus or of *Wolbachia* endosymbionts into the cytoplasm. Once released into a target population, these modifications can act to reduce one or more components of the mosquito population's vectorial capacity (e.g. the number of female mosquitoes, their longevity or their ability to support development and transmission of the pathogen). Some of the modifications under development are designed to be self-limiting, in that they will tend to disappear over time in the absence of recurrent releases (and hence are similar to the sterile insect technique, SIT), whereas other modifications are designed to be self-sustaining, spreading through populations even after releases stop (and hence are similar to traditional biological control). Several successful field trials have now been performed with *Aedes* mosquitoes, and such trials are helping to define the appropriate developmental pathway for this new class of intervention.

## 1. Introduction

Vector-borne diseases continue to plague us. The worst is malaria, transmitted by anopheline mosquitoes, killing many hundreds of thousands of people every year, mostly infants and children in tropical Africa [1]. The best existing methods of control—artemisinin-based drug treatment and mosquito control with chemical sprays and treated bednets—can reduce the burden of disease substantially, and can even eliminate the disease in some regions, but are not thought capable of globally eradicating the disease [2,3]. It is not even clear that current levels of efficacy can be maintained, given the likelihood of parasites and mosquitoes evolving resistance, and immunity waning as a result of partial control [3,4]. Dengue fever is another important mosquito-borne disease: caused by a virus originating in the jungles of southeast Asia, more than 100 countries have now been affected by dengue outbreaks, and the incidence of its most severe forms (dengue haemorrhagic fever and dengue shock syndrome) has increased over 500-fold since the 1950s [5]. Current methods of vector control can reduce dengue transmission, but even the most effective programmes seem unable to eliminate the disease. Other important (but less studied) vector-borne diseases include leishmaniasis, trypanosomiasis, Chagas disease, viral encephalitis, yellow fever, onchocerciasis and lymphatic filariasis [6].

Given the persistent burden of vector-borne diseases, there is substantial interest in developing qualitatively new methods of control. Much research is going into vaccine development for malaria and dengue fever, but both diseases present complexities to the vaccine designers that make success in the near term far from guaranteed [7–9]. For malaria, an indication of the difficulties comes from the Global Malaria Action Plan [10], which suggests that UD$250M will need to be spent per year for the next 15 years on malaria vaccine development (total $3.75B), and then $125M per year for the next 17 years after that, out to the year 2040. The example of yellow fever is also instructive: here is a mosquito-borne disease for which there has long been a highly effective vaccine, but still it kills tens of thousands every year, owing to the difficulties of vaccine delivery in many parts of the world [11].

It is in this context that many are working to expand the arsenal of vector control tools to include novel genetic approaches and heritable bacterial endosymbionts. A wide variety of such approaches have been proposed, and population and epidemiological models have shown that they could have a substantial effect, with excellent potential for sustained control and even elimination [12–20]. In this paper, I describe the various strategies being considered; review progress on their development; outline the potential value added by these approaches; and discuss some of the challenges for the future in bringing these technologies to the field. Most of the work focuses on *Anopheles gambiae* and *Aedes aegypti*, the most important vectors of malaria and dengue, respectively, and I henceforth refer to the target vector populations as ‘mosquitoes’, though similar approaches may in the future be developed for diseases transmitted by other insects. Much progress has been made in the past 10 years in developing what could prove to be truly transformational technologies (for other recent reviews, see [21–23]).

## 2. Classification of proposed strategies

Heritable approaches to control mosquito-borne diseases involve releasing a certain number of modified mosquitoes into a target population. Many different approaches have been proposed and are under development, and it is useful to highlight three ways in which they differ. First, there is the type of modification done to the released mosquitoes, in particular whether it is the introduction of one or more genes into the nuclear genome, or of a maternally inherited endosymbiont into the cytoplasm*.* Another key difference is in the intended effect on the target population, whether it is to reduce the numbers of female mosquitoes, their lifespan or their ability to support pathogen development and transmission to humans. All of these are different ways of reducing the vectorial capacity of the target population, defined as the rate at which a mosquito population successfully transmits new infections per infected human. In principle, other approaches for reducing vectorial capacity are also possible, such as targeting host seeking or feeding behaviour.

A third difference, perhaps the most important, is in the expected dynamics of the modification once introduced into a target population. There are many possibilities. Some constructs are self-limiting, having an inherent tendency to decline in frequency and disappear from the population. Repeated releases are necessary to maintain these constructs in the target population, and relatively large (i.e. inundative) releases will usually be necessary to have a significant epidemiological effect. Release of sterile males for population suppression is a clear example. By contrast, other constructs are meant to be self-sustaining, with an inherent tendency to increase in frequency in the target population over multiple generations and maintain themselves at a high frequency. Releases need occur only once or a few times, and can often be of relatively fewer mosquitoes (i.e. inoculative releases). The release of these self-sustaining constructs is more akin to traditional biological control (box 1). Yet other constructs will require relatively large releases to get established in the target population, but then are expected to maintain themselves at high frequency without further releases.

Box 1. **Antecedents.**

Although novel in many ways, heritable approaches to vector control do have similarities with some well-established methodologies.

Sterile insect technique (SIT). SIT programmes involve the release of large numbers of sterile males into a target population; mating of these males with native females leads to a reduction in the females' reproductive output, resulting in the depletion or elimination of the target population. SIT programmes have been highly successful against a number of agricultural insect pests, including New World screwworm fly (*Cochliomyia hominivorax*) in North America; Mediterranean fruit fly (Medfly; *Ceratitis capitata*) and other tephritid fruit flies in many countries around the world; pink bollworm (*Pectinophora gossypiella*) in the USA; and codling moth (*Cydia pomonella*) in Canada ([24] and references therein). SIT has also been used to eliminate tsetse fly (*Glossina fuscipes*), vector of trypanosomiasis, from Zanzibar.

Biological control. Self-sustaining heritable approaches have clear parallels with classical biological control programmes [25]. These involve releasing relatively small numbers of a natural enemy (predator, parasitoid, pathogen, etc.) to attack a pest species. The natural enemy propagates itself over a period of generations, increasing in frequency over time. The agent is thus self-spreading and self-sustaining, and the effects on the target population can be permanent (or at least long-lived). Implementation is relatively inexpensive and the benefit/cost ratios of some programmes have been more than 100 [26]. Classical biological control has been used to suppress over 200 species of invasive insects and 40 species of weeds in many countries around the world [25].

## 3. Self-limiting strategies

### (a) Wolbachia

In conventional sterile male release, males are irradiated and produce sperm that are able to fertilize an egg, but the embryos are aneuploid and die. Unfortunately, experiments with *Anopheles* mosquitoes have shown that irradiation dosages sufficient to sterilize male mosquitoes also cause significant reductions in their mating competitiveness [27,28]. *Aedes* mosquitoes may be more robust in this regard, and recent reports have shown promising results [29,30]. An alternative approach uses *Wolbachia* bacteria: these maternally transmitted endosymbionts somehow manage to kill embryos derived from the mating of infected males and uninfected females [31]. Therefore, infected males that are released into an uninfected population will act as sterile males—the ‘incompatible insect technique’ (IIT). This approach was used more than 40 years ago, before *Wolbachia* was understood to be the causative agent, to eliminate a population of *Culex quinquefasciatus* (a vector of lymphatic filariasis) from a village in Burma (Myanmar) [32]. More recent research has focused on *Aedes polynesiensis,* the primary vector of *Wuchereria bancrofti* lymphatic filariasis and a significant vector of dengue in French Polynesia. This species naturally harbours a strain of *Wolbachia*, but alternative *Wolbachia* strains have been introduced into laboratory populations by introgression from *Aedes riversi* or by micro-injection of cytoplasm from *Aedes albopictus* [33,34]. In each case, the resulting *Ae. polynesiensis* strains are bidirectionally incompatible with the natural strain—that is, when males of one strain mate with females of the other, the vast majority of progeny die. Males carrying the *Wolbachia* from *Ae. reversi* have been tested in both cage and small-scale field trials [35,36]. These latter trials showed that laboratory-reared and sorted males survive and competitively mate with indigenous *Ae. polynesiensis* females in the field, and motivate additional larger-scale trials. In addition, a *Wolbachia* strain from *Culex pipiens* mosquitoes has been transferred into *Ae. albopictus* [37,38], and the United States Environmental Protection Agency has recently approved IIT trials targeting the latter species in several locations in the USA (https://www.federalregister.gov/articles/2013/09/12/2013-22223/issuance-of-an-experimental-use-permit).

### (b) Transgenics

Transgenic approaches to creating sterile males have also been developed. In *Ae. aegypti*, a lethal positive feedback loop was created by putting a transcriptional activator called tTAV under the control of its own binding site; this construct produces high levels of tTAV protein which is toxic to the mosquito [39]. Importantly, the feedback loop (and lethal effect) is repressible by adding tetracycline to the diet, and in this way a homozygous strain can be maintained. Homozygous males that have been raised in the presence of tetracycline are released, mate with the wild females and all their (heterozygous) progeny die. The precise pattern and level of transgene expression can depend on where it is inserted in the genome, and as a result the lethality can occur early in the larval stage or at the larval–pupal transition. The latter may be more effective in suppressing mosquito populations, because early deaths release the rest of the population from density-dependent competition during the larval stage [39]. A particular strain of *Ae. aegypti*, OX513A, with an insert causing lethality primarily around the pupal stage, has now been tested in semi-field enclosure trials in Malaysia [40] and in small-scale field releases in Grand Cayman, Malaysia and Brazil, with releases large enough to demonstrate suppression of the target population in Grand Cayman and Brazil [21,41–43].

Recently, an alternative tetracycline-repressible system has been developed in *Ae. aegypti* in which only the daughters of released males die; the sons live to pass on the construct, so that some fraction of the granddaughters (and subsequent female descendants) of the released males will also die [44]. This is useful because the productivity of mosquito populations depends mostly on the females, and they are also the only ones to transmit disease. Population modelling shows that efficacy is particularly enhanced if males carrying two or more of these constructs are released [45]. This new construct makes use of control sequences from the *actin-4* gene that drive gene expression specifically in the indirect flight muscles of females; the result is females that are unable to fly in the laboratory (and are presumably unable to survive in the field), and apparently normal males. A particular strain with this construct, called OX3604C, performed well in both small and large laboratory cage trials, but less well in large field cages, though the cause of this difference is as yet unclear [46,47]. Preliminary steps have also been taken to establish the same system in *Ae. albopictus* (secondary vector of dengue) and *Anopheles stephensi* (primary vector of malaria in many urban areas of India) [48,49].

Another transgenic approach to creating sterile males was discovered serendipitously in *An. gambiae* when an endonuclease (I-PpoI) that cleaves a specific sequence in the rDNA repeat was put under the control of spermatogenesis-specific control sequences from the *β2-tubulin* gene [50]. Males that are heterozygous for this construct produce no viable progeny, apparently because the endonuclease is transmitted via the sperm into the zygote, where it cleaves the rDNA repeat on the maternally derived X chromosome, causing developmental arrest.

## 4. Self-sustaining strategies

**Figure 1.**
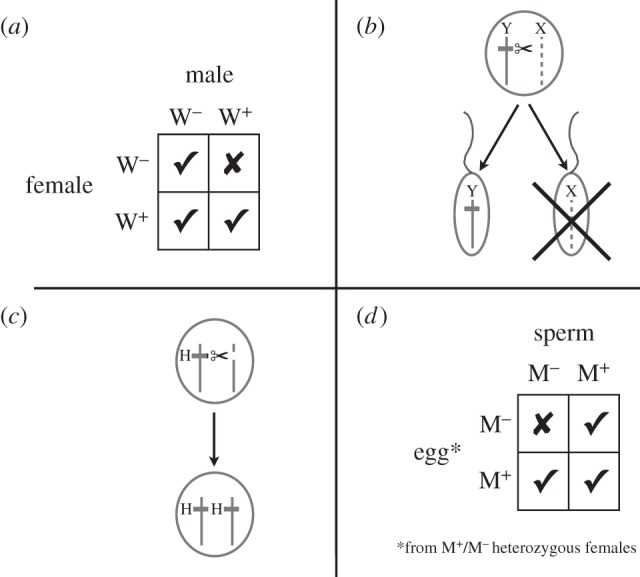
The logic of alternative gene drive systems. (*a*) Cytoplasmic incompatibility as induced by maternally transmitted *Wolbachia* bacteria. Infected females (W^+^) have an advantage because they can mate successfully with all males, whereas uninfected females (W^–^) can only mate successfully with uninfected males—matings with infected males produce few or no offspring. (*b*) Y chromosome drive, such as could be caused by an enzyme that cleaves the X chromosome at male meiosis, results in the majority of functional sperm bearing the Y chromosome, and a predominance of males among the progeny. (*c*) Homing endonuclease genes (H) cause the homologous chromosome to be cut and then get copied across during the repair process, converting a heterozygote into a homozygote. If the homing endonuclease gene is inserted into a host gene, then its spread through the population can lead to a population-wide gene knockout. (*d*) MEDEA elements gain a relative advantage because embryos from heterozygous (M^+^/M^–^) mothers die if they did not inherit the element (i.e. are homozygous M^–^/M^–^).

Self-sustaining approaches require a mechanism for spreading the desired trait through the target population and maintaining it at a high frequency. The most attractive such mechanisms use gene drive systems, in which traits spread because of a deviation from normal Mendelian inheritance [51,52] (figure 1). Many such systems exist naturally and have been discussed in the context of disease control; I focus on four for which there is at least some sort of laboratory proof-of-principle supportive data.

### (a) Wolbachia

*Wolbachia* strains can be used not only for self-limiting population suppression (above), but also for self-sustaining transmission control interventions. The cytoplasmic incompatibility that many strains cause, in which infected males effectively sterilize the uninfected females that they mate with, means that if *Wolbachia*-bearing females and males are released into a population in sufficient numbers, then the *Wolbachia* can spread through the population and be maintained at high frequencies. This self-spreading ability could, for example, be used to drive a *Wolbachia* strain that reduces adult lifespan through a population [53,54]. Because it is only the relatively old females that transmit disease, even small decreases in adult lifespan can have a large effect on vectorial capacity. Population modelling suggests there is a window of parameter space in which a life-shortening *Wolbachia* strain can significantly reduce dengue transmission and still spread [55,56].

More recently, attention has focused on the serendipitous discovery that *Wolbachia* infections can reduce vector competence [57–59]. Two different strains of *Wolbachia* from *Drosophila melanogaster, w*MelPop and *w*Mel, have been transferred to *Ae. aegypti*; the former proliferates more extensively within the mosquito body and, perhaps as a consequence, is more efficient at blocking dengue transmission, but it also imposes a more severe fitness cost upon the females, such that it is not clear it will be able to successfully establish in natural populations [53,58]. The *w*Mel strain also provides a significant block to viral transmission, yet imposes less of a fitness cost on the mosquito, and has been successfully established in two populations in northeast Australia [60,61]. *w*Mel has also been transferred to *Ae. albopictus*, where it also substantially reduces dengue transmission [62], and a *Wolbachia* strain has been transferred from *Ae. albopictus* to *An. stephensi*, in which species it both induces cytoplasmic incompatibility and provides some resistance to *Plasmodium falciparum* malaria infections [63].

### (b) Y drive

A number of nuclear gene drive systems are also under development. One approach, first suggested over 50 years ago, is to use a driving Y chromosome to reduce the number of females in a population [64–67]. Male mosquitoes contribute little or nothing except their DNA to the next generation, and so population productivity depends on the numbers and productivity of individual females. Therefore, biasing the sex ratio towards males can reduce or even eliminate target populations. As an added benefit, male mosquitoes do not bite people and transmit disease. In *Ae. aegypti*, some natural populations contain a segregation distorter that is closely linked to the male-determining locus and causes the latter to be transmitted to 80–90% of the progeny in crosses with sensitive strains [68,69]. Geographical surveys have shown that the distorter is present in some populations and not others, and that where it is present there is also substantial resistance, so sex ratios are not severely biased [69]. Little is known at the molecular level about how the distorter works, but cytologically it is associated with breakage of the X chromosome (i.e. the sex chromosome not containing the male-determining gene) during the first meiotic division [70]. In *An. gambiae*, Windbichler *et al*. [50] have shown that cleavage of the X-linked rDNA repeat during male meiosis results in the Y chromosome being transmitted to about 90% of the progeny, rather than the Mendelian 50%. As noted above, these progeny die, but if the two effects can be separated, such that the enzyme remains active in the testes but is inactive in the embryo, and the gene can be placed on the Y chromosome, then it may be possible to create an artificial driving Y. Recent modelling confirms this could have a substantial effect on mosquito population numbers and malaria transmission [15].

### (c) Population-wide gene knockouts with homing endonuclease genes

Homing endonuclease genes (HEGs) are parasitic or selfish genes that occur naturally in a variety of microbes [51,71]. As far as is known, they do not do anything useful to the host organism, and instead they spread and persist in populations because they encode an enzyme that, in cells heterozygous for the presence of the gene, cleaves the ‘empty’ chromosome. The HEG can then get copied across to the cut chromosome as a by-product of the chromosomal repair process, converting the heterozygous cell into a homozygote. Most naturally occurring HEGs are in the middle of self-splicing introns or inteins (sequences of amino acids that splice themselves out of proteins), and so do not disrupt the function of host genes, but they can be artificially inserted in the middle of a host gene in such a way as to knockout host gene function, yet still spread through the population. In principle, then, HEGs can act as reagents for population-wide gene knockouts [72]. Proof-of-principle demonstrations of this idea using a homing endonuclease from baker's yeast to knockout a transgene encoding a fluorescent protein marker and containing the cognate recognition sequence have been reported in both *Drosophila melanogaster* [73] and *An. gambiae* [74,75]*.* In the latter species, the HEG was shown to spread through small cage populations over multiple generations, knocking-out the marker gene as it did so.

To make a functioning gene knockout system, three components are needed: a target gene with the appropriate phenotype when knocked out; a homing endonuclease that recognizes and cleaves a specific sequence in the target gene; and control sequences that will turn on the HEG in the mosquito germline. Suitable targets may include genes involved in fertility, longevity, sex determination, host seeking or pathogen development or transmission. A recent review lists 34 *An. gambiae* genes for which there was some evidence from RNA interference (RNAi) knockdown experiments of reduced *Plasmodium* oocyst or sporozoite numbers in experimental infections [76,77]. Methods for engineering homing endonucleases to recognize new sequences have recently expanded [71,78,79], including assays in *Drosophila* [80]. Finally, control sequences from the *vasa* gene that activate transcription in the male and female *An. gambiae* germline have been characterized [81]. Homing endonuclease activity has also been demonstrated in *Ae. aegypti* [82].

### (d) Population-wide knock-ins

In principle, it may also be possible to reduce vectorial capacity by spreading through a mosquito population one or more genes that interfere with disease transmission. These strategies typically posit two components: one or more effector genes that act to block disease transmission and a gene drive system for spreading those effectors through the target population (the assumption being that the effectors will not give a significant selective advantage to the mosquito, and therefore will not spread of their own accord [52]). For malaria, a recent review lists 28 effector genes that interfere to some extent with parasite transmission [83], including anti-microbial peptides [84], single-chain antibodies [85], immune system activators [86,87] and peptides that bind to mosquito proteins (putative parasite receptors) in the midgut or salivary glands [88]. In the latter case, potential targets include some of the same molecules that are targets of transmission-blocking vaccines [89]. For dengue virus, which has an RNA genome, there have been promising results using both RNAi and ribozymes to attack the virus [90,91]. Many host factors have been described that may also be suitable targets for effector molecules [92,93]. It will be important to choose effectors that do not have large fitness costs for the mosquito, as otherwise non-functional mutants are likely to spread instead.

Multiple options are also being explored for the gene drive systems needed to spread these effectors though the target population. One possibility is an MEDEA element, which causes the progeny of heterozygous females to die unless they themselves inherit the MEDEA element [94]. In *Drosophila*, an artificial MEDEA element has been synthesized by combining a microRNA-based repressor of *myd88*, an important protein normally supplied by the mother into the embryo, with a zygotically expressed *myd88* gene that is not affected by the microRNA and supplies the missing protein [95]. This element spread rapidly through experimental cage populations: starting from an initial frequency of 25%, all individuals were found to contain the construct after just 10–12 generations. The challenge now is to develop a similar strategy for mosquitoes. In principle, other possibilities for driving effector genes into natural populations include using an engineered underdominance strategy [96] and/or using a driving Y chromosome or HEG to impose a selection pressure on a population, and then linking the effectors to a resistance gene [72,97].

## 5. Potential value added by these approaches

As currently envisaged, these various heritable approaches to vector control have a number of desirable features that motivate their continued development. Key attributes of the strategies include the following:

— they act to reduce transmission rates, not just morbidity and mortality, and thus can make an important contribution to the goals of disease elimination and eradication;
— they are widely applicable, able to act in diverse settings, whether hypo- or holoendemic, urban or rural, against indoor or outdoor biters, daytime or night-time biters, and can reach mosquito populations that are otherwise difficult to access;
— they provide area-wide control, and therefore protection without obvious biases relating to a person's age, wealth or education;
— they should be compatible with and complementary to other disease control measures, both current (e.g. chemical-based vector control) and under development (e.g. vaccines);
— they are taxon-specific in their targeting, thus reducing environmental risks; and
— they can be relatively easy to deliver and deploy (particularly the self-sustaining strategies), with little or no change required in how people behave, and as a result have the potential to be highly cost-effective.

As with any other form of pest or disease control, concerns about human and environmental safety must be incorporated into the design process. The possibility of resistance evolving in the vector or pathogen population must also be addressed, and steps taken to minimize this likelihood. Chemical approaches to controlling vector-borne diseases—drugs and insecticides—have been enormously useful over the past decades, saving many millions of lives, but for any one specific chemical it seems inevitable that resistance eventually arises, perhaps because exposures cannot be tightly controlled, and consequently are sometimes sublethal. It is unclear whether and how this dynamic will play out with heritable approaches to vector control, where exposures may be less variable. It is easy to imagine that mosquitoes, *Wolbachia* or dengue viruses might eventually evolve such that viral transmission is no longer blocked—but it is mostly a matter of speculation whether this would take years, decades, or longer. It is easy to model the spread of a gene conferring resistance to endonuclease-based Y drive, but unclear if and when such a mutation will arise in real populations. Similar uncertainties currently apply to the other self-sustaining approaches (resistance has rarely been a problem with conventional SIT programmes, giving some empirical basis for thinking it may not be a substantive problem for the newer self-limiting approaches). Combination therapy—giving patients multiple drugs simultaneously—has helped retard the evolution of resistance in many contexts, and much the same strategy should be explored in the context of heritable approaches to vector control—for example, using multiple endonucleases, or multiple effector molecules. Computer- and laboratory-based investigations will give useful information on the potential for resistance to evolve, but, as with anything else so new, some uncertainty will remain until constructs get to the field.

## 6. Moving forward

Heritable approaches to vector control represent a new platform for public health interventions, and consequently there has been a need to develop the appropriate frameworks for regulation, public consultation and community engagement. Experience with the development of novel drugs and vaccines has led to the concept of a product development pipeline from initial technology exploration, to field testing, and finally full implementation as a routine control measure. Much the same process should apply to the development of heritable approaches to vector control, though the criteria for advancing through the phases are novel, and are still being identified [98,99]. Useful lessons can probably be drawn from the closest antecedents to this technology, sterile male releases and biological control (box 1). Small-scale open releases of males with a dominant lethal, of males with an incompatibility-inducing *Wolbachia*, and of males and females with transmission-blocking *Wolbachia* have all been performed in *Ae. aegypti* or *Ae. polynesiensis* (reviewed in reference [21]), and there have been no reports of harm to human health or the environment. Lessons learned from these and related studies include the following:

— trials in large enclosures can be a useful intermediary step between small-scale laboratory studies and field releases [35,40,46,100,101];
— sometimes, it may not initially be apparent which body or bodies should be regulating the trials, but this does not preclude eventually finding a responsible agency [102];
— formal risk analysis procedures and appropriate experimentation to assess risks should precede release [103–105] (http://www.efsa.europa.eu/en/efsajournal/pub/3200.htm; http://bch.cbd.int/onlineconferences/forum_ra.shtml);
— local communities can give high levels of support to the trials once they are explained to them [106]; and
— in terms of efficacy, it will usually be appropriate to focus in the first instance on entomological outcomes; eventually, it will be important to track epidemiological outcomes, but this will require larger, more expensive studies [107,108].

Enormous progress has been made over the past decade on many fronts in the development of various heritable approaches to control vector-borne disease. While many challenges lie ahead in their continued gradual, step-by-step development, none appears insurmountable.
